# Parental Psychosocial Variables and Glycemic Control in T1D Pediatric Age: A Systematic Review

**DOI:** 10.1007/s11892-024-01566-y

**Published:** 2024-12-16

**Authors:** Vasco Costa, Bárbara Pereira, Susana R. Patton, Tânia Brandão

**Affiliations:** 1 William James Center for Research, Ispa-Instituto Universitário, Lisboa, Portugal; 2 Center for Healthcare Delivery Science, Nemours Children’s Health System, Jacksonville, FL USA

**Keywords:** Parental Psychosocial Variables, Children Glycemic Control, Type 1 Diabetes, Pediatric Age

## Abstract

**Purpose of Review:**

This review aimed to summarize the evidence regarding the relationship between parental psychosocial (e.g., fear of hypoglycemia, stress and family conflict) and glycemic outcomes in children between the age of 1–10 years old.

**Recent Findings:**

Type 1 Diabetes (T1D) in young children can be very complex to manage for their parents since they are the main individuals responsible for T1D tasks. Also, parental psychological adjustment impacts children’s glycemic outcomes.

**Summary:**

This systematic review was performed following the PRISMA guidelines. The search process was conducted in four databases from 2019 to 2024. From a total of 215 studies, 5 were included. We identified five studies that found direct associations between parental psychosocial variables and children's glycemic outcomes. These findings suggest a unidirectional perspective, evidencing the need to examine the longitudinal interplay between these variables. In sum, promoting parental psychological interventions may be fundamental for enhancing children’s glycemic outcomes.

## Introduction

The global prevalence of Type 1 Diabetes (T1D) in children and adolescents is estimated at 651,700 cases, with an annual incidence of 108,300 cases in this age group [1]. Worldwide, the five countries with the highest number of cases of type 1 diabetes by the age of 20 were India (282.832 cases), United States (170.408 cases), Brazil (112.240 cases), China (66.040 cases) and Russia (58.338 cases) [1]. In 2022, it was estimated that T1D affected 672 children aged 0–9 years in Portugal, and 1,184 cases were recorded among those aged 10–14 years [2]. Addressing T1D during early childhood is critical, as children have a heightened insulin sensitivity and unpredictable eating behaviors, among other factors, that often leads to significant glycemic fluctuations and severe hypoglycemic episodes [3, 4]. Around the age of 10, involving children in diabetes tasks also may be essential for parents, in order to start the process of transitioning responsibility for managing T1D [5]. In this sense, there are multiple challenges in diabetes management for parents of young children, to achieve clinical recommended levels of HbA1c and glycemic control [6, 7]. Carrying out these tasks effectively and regularly is essential to ensure that the levels of HbA1c remain stable and to avoid complications (e.g., strokes, heart attacks, kidney disease, eye issues, or even cognitive decline) [8–11].

There are several psychosocial variables that influence parents’ functioning which and may reflect on how they care for their children with T1D [12]. Psychosocial variables can be defined as the fusion between psychological and social factors. Mainly the psychological variables can be comprised of different factors, such as distress, anxiety, stress and depression symptoms, which also are divided by trait (e.g., personality traits and quality of life) and state dimensions (e.g., happiness and humor) [13]. On the other hand, social support, family environment and dynamics are part of the social factors [14].

Research has focused on how parents cope with this responsibility, revealing that many experience significant levels of diabetes distress (DD), anxiety, stress, and depression, which are attributed to the daily demands of managing T1D [15, 16]. The associations between these parental psychological factors (e.g., anxiety and depression) and their children´s glycemic outcomes are well documented [4–6]. We will now focus on the results concerning diabetes-specific psychological factors, such as diabetes distress and fear of hypoglycemia. Diabetes distress (DD) is a common psychological concern among individuals with diabetes and in their caregivers. DD can be defined as the emotional response to living with and caring for someone with T1D, and associated with fulfilling the challenging daily tasks of diabetes management as well as worrying about short- and long-term consequences (e.g., PAID-PR scale- item 8 and 18: “I feel angry when I think about my child having/living with diabetes” and “I feel “burned out” by the constant effort to manage diabetes”), of managing diabetes [16]. According to a recent investigation, parent and child DD positively associate with HbA1c levels. Also, parent DD, children’s insulin pump use, and children’s T1D duration, all combined, significantly explaining (18.2%) children’s HbA1c levels variation [16], reflecting the importance of developing psychological interventions targeting parental diabetes distress.

Fear of hypoglycemia is also frequently present among parents of young children with T1D due to glycemic variations, related to many variables, such as carbohydrate ingestion, lack of insulin, psychical activity, and others [17, 18]. However, the most common parental fear is related to hypoglycemia, which can be caused essentially by an inappropriate amount of insulin administration or lack of food intake [18]. Hypoglycemia can be dangerous as it may cause minor symptoms, such as confusion and sweating, as well as more serious outcomes, such as seizure, coma, or death. Data suggest that hypoglycemia period can be stressful for parents to manage [19]. Also, there is evidence that greater parental fear of hypoglycemia may be linked to higher levels of HbA1c, which is relevant to clinical management of T1D [19].

Hypoglycemia episodes can occur at any time of the day, although some of the most worrisome times can be at night. All glucose excursions (hyper- or hypoglycemia) that occur during sleep can have a direct impact on parents' sleep quality and consequently can influence how they care for their child and manage their child’s glucose levels on daily basis [20]. To prevent complications, parents usually set several alarms during the nighttime to measure or monitor children's glycemia. Some caregivers report sleeping next to their child to monitor their child’s glycemia. These common strategies can lead to parental, and child sleep deprivation and increases the possibility of developing physical and psychological complications over time (e.g., memory deterioration, higher levels of anxiety and depression) [21].

Related to the complex management of their child’s T1D, parents’ ability to cope and regulate emotions plays an important role in their psychological adjustment. Evidence shows [22] that high levels of distress are associated with reduced self-regulation in caregivers. Also, there is evidence that parental stress and greater levels of parent depression and anxiety related to T1D management predict higher child glycemic levels [23]. In this sense, it is important for parents to monitor their feelings and emotions and seek assistance when they identify negative feelings [24, 25]. The family and social support can also be very helpful at mitigating the constant psychological challenges of caring for a young child’s with T1D. The literature suggests that social support can enhance a parents’ ability to manage their child’s T1D and general self-care, which can indirectly impact their child’s glycemic control [26]. However, there is also evidence of an association between higher family conflict and lower T1D engagement and higher child glucose levels [27] in families, as well as an association between lower levels of parental marital relationship satisfaction and child glycated hemoglobin (HbA1c) [28], suggesting the constant and complex impact of T1D management on children and caregivers can influence ruptures in the family environment.

## The Present Systematic Review

Researchers have examined how parental psychosocial factors influence children's glycemic outcomes in T1D. Studies indicate that alleviating parental burden and enhancing caregivers' cognitive and emotional resources can positively affect a child’s glycemic control [29]. On the other hand, poor glycemic control can exacerbate diabetic distress (DD) and increase the fear of hypoglycemia (FH) [30]. A previous scoping review was conducted, including studies from 2004 to 2019, and providing a broad overview of these associations [31]. This scoping review included studies recruiting children with a mean age of 10 years old and studies that reported on child HbA1c as the sole biologic outcome. However, an updated systematic review can provide a more comprehensive synthesis of the current findings, highlighting the complex relationships between these variables and setting a focused agenda for future research. We assert an updated systematic review is also needed because it has been five years since the last scoping review, and significant research has been conducted in this area, especially following the COVID-19 pandemic, which introduced additional challenges and heightened stress levels for families [32]. Moreover, it is suggested that the virus may have played a pivotal role in increasing the prevalence of T1D in very young children [32]. Therefore, targeting this age group and analyzing parents' psychosocial factors and their relationships with their child's glycemic outcomes is crucial to verify the research advances. Our methods for this systematic review are in some ways like those of Brito and Remor [31]. We adopted similar inclusion criteria and aims, focusing on the experiences of families of very young children with T1D. A notable difference in our methods is the choice to exclude studies recruiting parents of children greater than 10 years old. Our methods also include studies reporting child HbA1c or continuous glucose monitoring data as biologic outcomes.

## Method

The present systematic review (SR) followed the Preferred Reporting Items for Systematic Reviews and Meta-Analyses (PRISMA) Guidelines [33].

## Literature Search and Inclusion Criteria

To identify relevant papers, searches on EBSCO host, PubMed, SCOPUS, and Web of science databases were undertaken. The database searches included a restriction date between January 2019 and till March 4th, 2024, to summarize new research not included in the previous scoping review [31].

**Fig. 1:**
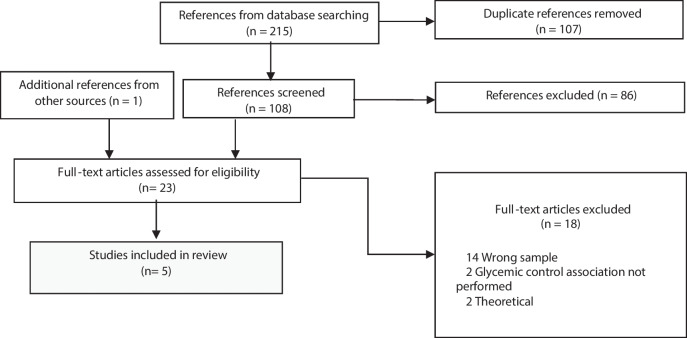
PRISMA Flow Diagram

The following combined key terms were used: (diabetes AND type 1) AND (parent* OR mother* OR father* OR famil* OR caregiver*) AND (longitudinal OR cross-sectional) AND (adjustment or adaptation OR well-being OR distress OR stress OR anxiety OR attachment OR “quality of life” OR “quality of sleep” OR depression OR “fear of hypoglycemia” OR “social support” OR “emotion regulation” OR “family conflict*”) AND (child* OR kid OR kids) AND (“glycated hemoglobin” OR “hemoglobin A1c” OR HbA1c OR “glycemic control” OR “metabolic control “ OR “glycemic outcome*”). While numerous psychosocial variables could have been included in our search, it was not feasible to incorporate all of them. Therefore, we selected terms based on previous studies that explored parental psychosocial variables, as well as search terms used in a prior scoping review [31]. Studies eligible for inclusion needed to comply with the following criteria: 1) Studies with parents (e.g., couples or single parental families) of children with T1D aged 1 to 10 years old; 2) Cross-sectional and longitudinal studies; 3) Quantitative articles or Mixed method (e.g., only quantitative data reported); 4) Empirical peer-reviewed articles; 5) Written in English, Portuguese or Spanish. 6) Parental psychosocial variables, such as distress, fear of hypoglycemia, social support, quality of life, quality of sleep, attachment, anxiety, depression, stress, and emotion regulation; 7) Glycemic outcomes, such as HbA1c, CGM (Continuous Glucose Monitoring), and capillary blood samples. The exclusion criteria were: 1) Parents of children over 10 years old; 2) grey literature, thesis, unpublished works, or other systematic/scoping reviews; 3) qualitative studies and randomized and nonrandomized controlled trial. The flow diagram of the study selection process is presented in Fig. 1.

## Study Selection, Data Extraction, and Study Coding

Two researchers independently coded the fully read documents selected (Fig. 1). A third author resolved any disagreements through consensus.

First, studies from several databases were extracted and duplicate articles were excluded by the two investigators. Other sources (e.g., google academic) were analyzed to expand our search. Using titles and abstracts, we identified those studies which met the inclusion criteria. Once this task was completed, the selected articles were fully analyzed by the same two researchers. In case of disagreements a third author was consulted to make the final decision. Finally, articles that fulfilled all requirements were selected. In total we extracted 215 articles. The removal process regarding duplicated studies took out 108 articles, leaving 109 articles (one additional reference from google academic was added). From these 109 articles, we excluded 86 studies. In this sense, the initial screening resulted in 23 references for full-text eligibility review. After full-text screening 18 studies were excluded mainly due to the wrong sample, wrong design, or because there was no association to child glycemic control.

In the end, a final sample of 5 papers met our requisites. The following information about each study was collected: 1) Author, year, country and language of the publications; 2) Study objectives; 3) Children’s age and glycemic variables; 4) Main results. Information was coded from all articles, and the accuracy of that coding was checked by the two authors. Disagreements were also resolved by consensus and discussion with a third member.

## Quality Appraisal

Studies quality appraisal was analysed using the Mixed Methods Appraisal Tool (MMAT, version 2018) [34]. The MMAT comprises two screening questions and five items for assessing the methodological quality of qualitative and quantitative studies. Also, the representativeness of participants, appropriateness of measurements, quality of the results and appraisal of confounders are also analysed. Each of the criterion is rated as “yes,” “no,” or “can’t tell”. The “yes” is rated with 1, and “no” and “can't tell” is rated with 0, yielding a total score for each study ranging between 0 and 5 points.

## Results

### Study Characteristics

#### Sample

Five studies targeted parents of children until the age of 10 and the sample size of the studies ranged from 54 [35] to 128 [36]. Studies were composed mostly by mothers 83.7%−87.8% [36–39]. The studies were published between 2020–2021. Four studies were conducted in US [36–39] and the other one was performed in Egypt [35]. In terms of design, one study used a cross-sectional quantitative design [35] and four studies used a longitudinal quantitative design [36–39]. According to MMAT quality appraisal tool, four studies earned three out of five points [35, 37–39] and one study [36] earned two out of five points. A description of all included studies and their main results are included in Table 1.

**Table 1:** Studies characteristics

Article	Year	Country	Design	Article language (EN/PT/SP)	Study objectives	Children age and glycemic variables utilized	Main results
Case et al. [37]	2021	US	Longitudinal Study	EN	To investigate the relationship between family conflict, parental T1D engagement and glycemic outcomes	5–9 *(M* = 7.8, *SD* = 1.3 years) HbA1c	A negative correlation was evident between diabetes family conflict total scores and their parents’ diabetes self-management scores. Parent diabetes self-management scores remained consistent at time 2) but displayed a notable increase at time 3. An observable impact of family conflict on self-management scores emerged, with each unit increase in conflict predicting a 0.14 decrease in parent-reported self-management scores Furthermore, a significant temporal effect on children's HbA1c levels was observed. HbA1c values were notably elevated at time 2 and time 3 compared to time one A statistically significant association was identified between increasing diabetes family conflict total scores and escalating children HbA1c levels
Elhenawy et al. [35]	2021	Egypt	Cross Sectional Study	EN	To assess the impact of the COVID-19 pandemic and lockdown on glycemic control in Egyptian youth with Type 1 Diabetes and to identify lockdown-associated factors influencing glycemic control	0–5 years (n = 10), 5–10 years (n = 44). HbA1c	Severe stress was notably higher among caregivers of infants and toddlers with diabetes. A significant positive correlation was found between perceived stress scale and glycemic control (HbA1c), after lockdown. Although nonsignificant, HbA1c improved in young children while it significantly increased in school-age children and adolescents. Nearly half feared hospital admission and infection, with – > 29% feeling more susceptible. There was a significant positive correlation between perceived stress scale and pre-lockdown HbA1c. Most expressed concern about medical supply shortages. Perceived stress levels during lockdown, suggested with – > 60% of parents reporting moderate stress and – > 40% reporting severe stress
Patton et al. [38]	2021	US	Longitudinal Study	EN	To examine HbA1c patterns and identify parental psychosocial predictors of HbA1c trajectory in young children, in T1D recent-onset period	*M* = 7.50, *SD* = ± 1.3 (5 to 9 years old) HbA1c	Significant associations between parental psychosocial factors and children's HbA1c metrics were found. Parents' HFS-P Behaviour scores negatively correlated with HbA1c above 7.5% (58 mmol/mol). Children’s HbA1c slope and density, predicted parental diabetes distress immediate scores. Conversely, diabetes family conflict total scores were positively linked with HbA1c above 7.5% (58 mmol/mol) and diabetes distress theory scores positively correlated with children's HbA1c slope and density Higher HFS-P Behaviour and diabetes family conflict indirect scores increased the likelihood of a high HbA1c trajectory, while higher diabetes family conflict direct scores and diabetes distress immediate scores decreased it Elevated diabetes family conflict scores predicted a high stable trajectory, whereas higher parental fear of hypoglycemia scores reduced this likelihood Elevated diabetes family conflict scores were predicted a high stable trajectory, whereas higher parental fear of hypoglycemia scores reduced this likelihood
Stanek et al. [36]	2020	US	Longitudinal Study	EN	To verify the influence of stressful life events in newly diagnosed school-age children and their psychological stressors	*M* = 7.4, *SD* = ± 1.3. HbA1c and CGM	Associations were observed between reduced family income and heightened child HbA1c levels at 9 and 12 month assessments. A decline in self-monitoring blood glucose (SMBG) frequency was noted at 12 month interval among those experiencing a decrease in income. Notably, there was no significant difference in continuous glucose monitoring (CGM) usage between individuals affected by reduced family income and those unaffected Parental job changes at the 9 month evaluation were linked with increased child HbA1c levels. Changes in school attendance were also associated with elevated child HbA1c levels and reduced SMBG frequency at the 12 month assessment. However, no significant difference in CGM usage was observed between individuals experiencing changes in school attendance and those who did not. Notably, no significant associations were found between marital status or health changes and child HbA1c levels throughout the study
Youngkin et al. [39]	2020	US	Longitudinal Study	EN	To evaluate parental fear of hypoglycemia (FH) and CGM adoption rates at T1D recent-onset and explore CGM impact on parental FH	*M* = 7.5, *SD* = ± 1.4 (range = 5—9) CGM and HbA1c	The mean total score on the Hypoglycemia Fear Survey-Parental (HFS-P), indicated a moderate level of apprehension among participants. Subsequently, between time 1 and 2, there was a notable decrease in reported HFS-Behaviour scores among caregivers. However, there was no discernible alteration in HFS-Behaviour scores between time 2 and 3. At time 1, parents displayed an average HFS-Worry score which significantly increased between time 1 and 2, remaining unchanged thereafter. A significant relationship was identified between the initiation of CGM between time 1 and 2 and parents' HFS-Behaviour scores at time 1 Conversely, there was no association between parents' HFS-Behaviour scores at time 1 and CGM commencement between time 1 and time 2. Similarly, no association was observed between parents' HFS-Worry scores at time 1 and CGM onset between time 1 and 2. However, a significant correlation was noted between CGM initiation between time 2 and 3 and parents' HFS-Worry scores at time 1 Finaly, average HbA1c from time 1 to time 3 increased significantly (T1 = 7.6%; T2 = 8.1%; T3 = 8.3)

Results extracted from the five studies, identified that, all five studies examined associations between parent psychosocial variables (FH, DD, parental stress, family conflict and parental engagement) and child outcomes, which highlights the crucial role of caregivers on their children´s glycemic levels and T1D management.

#### Parental Psychosocial Variables and Children Glycemic Outcomes

Five studies aimed to analyze the interaction between parental psychosocial variables and young children’s glycemic outcomes [35–38]. These positive health outcomes also may decrease the probability of diabetes complications for children.

Case et al. [37] in a longitudinal study with 127 US parent–child dyads (children aged 5–9 years, and newly diagnosed with T1D), found a negative correlation between family conflict and parent engagement in their child’s T1D treatment. Conversely, a positive correlation was found between family conflict and children’s HbA1c levels. Throughout the three assessment periods, only one change in family engagement was observed, happening at the third assessment point, and coinciding with a significant increase in children’s HbA1c levels between the second and third assessment points.

Elhenawy et al. [35] using a sample of 54 Egyptian parent–child dyads (ages 0 to 5, n = 10), ages 5 to 10, n = 44), examined variables associated with lockdown and their impacts on children’s glycemic outcomes. Results highlighted that parental stress, both before and after confinement, was positively correlated with glycemic control (HbA1c).

Patton et al. [38] conducted a longitudinal study with 106 parent–child dyads in the US (children aged 5–9, 58 girls and 48 boys) to examine HbA1c trajectories in children with newly diagnosed T1D and their association with parent-reported psychosocial factors. On average, children's HbA1c levels increased over time, showing significant variability between early and later stages of the new diagnosis period. Higher parental FH predicted a high stable or intermediate increasing HbA1c trajectory in children. Immediate parental distress negatively correlated with HbA1c slope and density, while family conflict correlated with elevated HbA1c levels in children. Finally, long-term parental distress positively correlated with HbA1c in children.

Stanek et al. [36], in a longitudinal study with 128 US parent–child dyads (children aged 5–9 years), assessed the impact of stressful events in recently diagnosed children with T1D and verified psychological stressors among their caregivers. Specifically, they found that decreased family income predicted higher child HbA1c levels at 9- and 12-months and less frequent self-monitoring of blood glucose (SMBG) at 12-months, without affecting children’s glucose levels as measured via CGM). Parental job changes, at 9- months, and child school changes at 12-months were also associated with higher HbA1c and less frequent SMBG, with no associations to daily glucose levels based on CGM.

Youngkin et al. [39] analyzed the relationship between FH and the initiation and use of CGM, in 96 US parent–child dyads (children aged 5–9) with recent-onset T1D. The average HbA1c from time 1 to time 3 increased significantly (T1 = 7.6%; T2 = 8.1%; T3 = 8.3). Regarding time 1 and time 2, 31 children initiated CGM therapy, with another 17 children starting between time 2 and time 3. According to the HSF-P total scores, at time 1, the parents showed moderate levels of FH. At time 2 these scores increased significantly and stabilized at time 3. A significant association was verified between CGM initiation from time 1 to time 2 and parents’ time 1 HFS-Behavior scores. In the other hand, parents’ time 1 HFS-Worry scores were only significantly linked to CGM starts from time 2 to time 3. Parents of children that began CGM between time 1 and time 2 had higher T1 HFS-Behavior scores and experienced greater decrease of these scores. For CGM initiation between time 2 and time 3, parents had higher T1 HFS-Worry scores. In sum, the results highlight the pertinence of the glycemic control trough CGM, after 6 to 12 months of T1D diagnosis, resulting on reduce parental FH.

## Discussion

The aim of this systematic review was to examine the relationships between parental psychosocial variables and children’s glycemic outcomes. Based on the inclusion criteria for this systematic review, five studies were included. Consistent with the previous [31] scoping review, it appears this literature base continues to offer mixed and inconclusive evidence regarding the associations between parental psychosocial variables and child glycemia. Though four of the studies used a longitudinal design, which can help to establish temporal associations among the variables, none of these studies employed a design that might allow one to infer causation. Nevertheless, longitudinal studies are still under-represented (e.g., since they take more time and resources to be carried out) and, also applying sophisticated statistical models and analysis (e.g. crossed lagged panel model), could provide crucial data. In this sense, understanding the complex relationship between these constructs, may reveal dynamic interactions that could have critical implications for both the development of new treatment approaches and supportive healthcare policies.

Results from Case et al. study [37] demonstrated a significant association between increasing levels of family conflict, parental engagement, and increased children HbA1c levels. It is possible that heightened family conflict during this crucial early stage of T1D may compromise parental engagement thereby relating to children’s HbA1c levels. Similar results have been observed in adolescent samples [40, 41]. Also, this association is consistent with Patton et al. [38], which reported a strong positive association between parent-reported family conflict scores and higher child HbA1c and Jaser et al. [42], which associated maternal depression with child glucose.

Regarding parental stress, both before and after the Covid-19 pandemic, it appears stress was positively associated with child glucose levels and HbA1c levels as reported by Elhenawy et al. [35]. Other studies reveal a similar association between elevated parent stress and T1D management difficulties in families of children living with T1D [43–46]. Moreover, in a longitudinal study, Stanek et al. [36] found parental perceptions of stressful events such as a decrease in family income, school changes, and parental job changes associated with higher levels of child HbA1c. Additionally, they found depression scores, family conflict, and avoidance coping associated with the report of stressful events (e.g., at least one event) indicating that the occurrence of stressful life events can relate to parent psychosocial outcomes as well. Longitudinal studies need to apply a more comprehensive framework to incorporate reciprocal associations, considering mediators and moderators.

Specific to DD, a longitudinal study [38] showed a positive association between parental long-term DD and children's HbA1c trajectories in the new-onset period. This study also suggested greater probability of children following a high stable or intermediate increasing HbA1c trajectory compared to a low stable trajectory when parents report higher FH levels. Taken together, these studies may be among the first to establish a temporal association between parent psychosocial variables and child glycemia.

Youngkin et al. provided the only evaluation of the association between parent psychosocial variables and CGM uptake [39]. Their results suggested a relationship between parents’ FH levels near to the time of T1D diagnosis and uptake of CGM in the first 12 months post-diagnosis. Notably, while starting CGM appeared related to a decline in parent reported use of potentially maladaptive hypoglycemia avoidance behaviors in the new-onset period, it did not appear to impact parental worries regarding FH, potentially reinforcing the existence of an emotional quality to FH that may not be lessened with a device.

As for the quality of the studies included none of the studies attained a classification above three in the MMAT tool. The reviewed studies, while providing valuable insights, share some limitations that impact their overall quality and generalizability. A primary concern is the lack of diversity in the samples. Many of these studies predominantly feature non-Hispanic White and higher income participants, which restricts the applicability of the findings to more diverse racial, ethnic, and socioeconomic groups (e.g., Patton et al. [38]; Stanek et al. [36]; Youngkin et al. [39]). The homogeneity in the sample composition raises significant concerns about the generalizability of the results to broader populations (e.g., most of the studies were composed mostly by mothers, which limits the generalization for fathers). Selection bias is another pertinent aspect that must be addressed. The reliance on non-probability sampling and the potential differences between clinics contribute to this bias (e.g., Youngkin et al. study [39]. Additionally, the absence of detailed reasons for non-participation and targeted efforts to ensure demographic diversity suggest that the studies may not fully represent the intended populations. Finaly, the control for confounders, however addressed through good practices such as comprehensive baseline data collection and regular follow-ups, remains an area for improvement. The current statistical methods would benefit from the incorporation of more robust statistical techniques. Advanced regression models, for example, could better account for multiple confounders simultaneously in some of the included studies (e.g., Stanek et al. study [36]), providing clearer and more accurate insights into the relationships in focus. In sum, the demographic limitations of the samples, coupled with selection biases and insufficient control for confounders, may limit the generalizability of the findings, although the findings of the five studies are reliable, but must be interpreted and generalized with caution.

Finally, these results suggested that could be important to apply a socio-ecological perspective as suggested by Stanek et al. study [36]. In this sense, to implement a socio-ecological perspective, it is crucial to have robust empirical support, regarding a theoretical model that integrates factors at different levels [47]. Then, the use of several mediators and moderators (e.g., sociodemographic factors), can be important to explain the relationship between parents’ psychosocial factors and children’s glycemic outcomes. Finally, concerning the implications, providing a holistic understanding of how different factors interact, could pave the way to develop tailored interventions regarding these moderators and mediators, and may provide crucial theoretical evidence for the future investigations.

## Future Research, Implications and Strengths

All studies adopted a unidirectional perspective, highlighting a significant gap in the literature. Also, none of the four longitudinal studies included in the review, addressed the interplay and reciprocal associations over time between parental psychosocial variables and children’s glycemic outcomes, suggesting a need for future investigations in this area [31]. Future studies must address the existing gaps in the literature through the development of more longitudinal studies, using robust theoretical models and powerful statistical designs to establish advanced associations, as well as employing a socio-ecological perspective, considering the influence of life stressors. Furthermore, there is a need for studies that apply an expanded conceptualization of parent psychosocial variables, such as attachment, parenting styles and social support (e.g., extending beyond FH, DD, stress).

To address quality of future research, several recommendations may be considered. Future studies should prioritize recruiting more diverse samples to better reflect the target population. Also, implementing probability sampling strategies and recruiting from a broader range of clinics or community settings would enhance representativeness. Additionally, incorporating advanced statistical techniques to control for multiple confounders could be fundamental. Addressing these limitations, in future research may provide a more comprehensive and generalizable insights, ultimately contributing to a better understanding of the studied phenomena and improving the quality of scientific evidence.

The current systematic review highlighted the association between parental psychosocial variables and glycemic control suggesting important information for developing new research, in the context of parents of young children till the age of 10 years old, with T1D. Additionally, 4 studies were conducted in the US. Although of the generalization challenge, these findings will certainly be an essential starting point to discover and replicate in other countries and families.

The results regarding FH, DD, parental stress, family conflict, parental engagement, and their impact on children’s glycemic control, can serve as a catalyst for raising awareness among healthcare organizations and policymakers regarding the value of psychological research. Also, these findings may help providers to better understand the relationship between the parental psychosocial factors, and children’s glycemic outcomes, which consequently, could improve their approach on counselling since these relevant variables can be target in interventions. Based on the findings of this review, psychological interventions, such as REDCHIP (Reducing Emotional Distress for Childhood Hypoglycemia in Parents) [48] or CARES (Cognitive Adaptions to Reduce Emotional Stress) [49], may be beneficial to improve parents’ psychosocial variables and, consequently, children’s glycemic control. Thus, an important strength of this systematic review was the inclusion of articles written in English, Portuguese or Spanish.

## Limitations

Concerning the findings of this review, several factors need to be considered. First, the exclusion of grey literature may have introduced publication bias, as valuable insights from unpublished sources or non-peer-reviewed materials could have been overlooked. Furthermore, the exclusion of qualitative studies limited our ability to capture nuanced perspectives and experiences related to parental psychosocial variables and children's glycemic outcomes. Moreover, by restricting the age range of the participants, we may have restricted the range of potential studies we could have included. Also, the lack of diversity in the sample studies, particularly in terms of country, race, socioeconomic status, and ethnicity. Finally, many important variables, such as attachment, sleep quality, coping strategies, social support, and emotion regulation, were not founded in contrast to findings from the previous scoping review [31].

## Conclusions

Our findings and the current literature present a unidirectional perspective regarding parental psychosocial factors and children’s glycemic outcomes, as well as important mediators/moderators related to this relationship may be considered for future research. Also, longitudinal designs with sophisticated statistical analyses could possibly pave the way for evaluate reciprocal associations, over time, concerning these constructs. Additionally, interventions targeting parental psychosocial variables could play a pivotal role on improving their quality of life and consequently may enhance their children glycemic control.

## Data Availability

No datasets were generated or analysed during the current study.
